# Psychosocial distress after radical prostatectomy, radical cystectomy, or (partial) nephrectomy – a comprehensive analysis of 4,290 German cancer patients during the COVID-19 pandemic

**DOI:** 10.1007/s11764-024-01644-w

**Published:** 2024-07-10

**Authors:** Henning Bahlburg, Patricia Rausch, Karl Heinrich Tully, Sebastian Berg, Joachim Noldus, Marius Cristian Butea-Bocu, Burkhard Beyer, Guido Müller

**Affiliations:** 1 Department of Urology, Marien Hospital Herne, Ruhr-University Bochum, Hölkeskampring 40, 44625 Herne, Germany; 2 Center for Urological Rehabilitation, Kliniken Hartenstein, Bad Wildungen, Germany

**Keywords:** Psychosocial distress, Prostate cancer, Bladder cancer, Kidney cancer, COVID-19 pandemic

## Abstract

**Aim:**

To evaluate and identify predictors of psychosocial distress (PD) in patients after surgical treatment for prostate cancer (PC), bladder cancer (BC), or kidney cancer (KC) during the COVID-19 pandemic in a large, multi-institutional cohort.

**Material and methods:**

Patients undergoing inpatient rehabilitation (IR) after radical prostatectomy (RP), radical cystectomy (RC), or (partial) nephrectomy in one IR center in 2021 were included. PD was evaluated by the Questionnaire on Stress in Cancer Patients (QSC–R23) at the beginning (T1) and the end (T2) of IR. Regression analyses were performed to identify disease-specific predictors for high PD.

**Results:**

A total of 4,290 patients (3,413 after RP, 563 after RC, 314 after (partial) nephrectomy) were included in this study. Median PD decreased significantly during IR across all tumor entities (each *p* < 0.001). The number of PC and BC patients suffering from high PD decreased significantly (each *p* < 0.001), but not in KC patients (*p* = 0.310). Younger age independently predicts high PD in all three malignancies, while additionally positive surgical margins (*p* = 0.016), ileal conduit (IC; *p* < 0.001), and nephrectomy (*p* = 0.032) independently predict high PD in PC, BC, and KC patients, respectively. During the Covid-19 pandemic the demand for individual psycho-oncologic counseling increased significantly in PC (*p* = 0.03) and KC (*p* = 0.001) patients.

**Conclusion:**

Younger age independently predicts high PD in the three main urological malignancies. Positive surgical margins in PCa, IC in BCa, and nephrectomy in KC are disease-specific independent predictors for high PD in the early period after surgical treatment.

**Implications for Cancer Survivors:**

Disease-specific predictors for high PD may help clinicians identify patients at risk and may guide timely referrals to psycho-oncologic counseling in the early period after uro-oncologic surgery.

## Introduction

Psychosocial distress (PD) is highly prevalent among cancer patients and may not only significantly impact mental health but also quality of life and is therefore considered the “sixth vital sign” in cancer patients in addition to the established vital signs blood pressure, heart and respiratory rate, body temperature, and state of consciousness [1]. Between 30 and 75% of cancer patients develop depression, anxiety, or an adaptive disorder during therapy [2–5] with fear of disease progression being a driver of high PD [6]. Current German guidelines strongly recommend continuous psycho-oncological support as a standard component of cancer treatment [7]. Furthermore, patients treated in certified cancer centers routinely undergo perioperative psycho-oncologic counseling. Nonetheless, up to 89% of patients lament insufficient psycho-oncological care during and after cancer treatment [8].

About 30% of prostate cancer (PC) patients suffer from psychosocial distress [2]. As recently demonstrated by our group analyzing PD in 587 patients after radical prostatectomy (RP) from a single German high-volume institution, age ≤ 69 years and tumor stage ≥ pT3 are independent predictors for high PD. However, PD decreased significantly during inpatient rehabilitation (IR) [9].

Previous studies by our group identified age ≤ 59 years as an independent predictor for high PD in the early period after radical cystectomy (RC) for bladder cancer (BC) [10]. However, even before RC, up to 35% and 45% of patients show signs of depression and suffer from high PD, respectively [11, 12].

The data on PD in patients with localized kidney cancer (KC) undergoing (partial) nephrectomy is scarce when compared to other urological malignancies. As demonstrated by Draeger et al., about 27% of KC patients suffer from elevated PD [13]. Bergerot et al. showed an association of female sex, younger age, non-clear cell histology, and recurrent disease with PD [14]. A cross-sectional single-center study by Pastore et al. showed significantly higher levels of anxiety and depression in patients with kidney cancer [15]. Furthermore, surveillance for small renal masses is associated with elevated distress levels [16]. So far, most studies have focused on patients with advanced KC [17], included only a small number of patients [15], or patients with localized and metastatic disease [13, 14], or assessed only anxiety or depression [18] resulting in a paucity of data on PD in patients with localized KC undergoing (partial) nephrectomy.

During the COVID-19 pandemic both elevated levels of distress, anxiety, and depression and higher rates of suicide and post-traumatic stress disorder were detected among the general population [19]. This multi-institutional study aims to analyze PD among the three major genitourinary malignancies in patients undergoing IR during the COVID-19 pandemic, compare results to data obtained before the pandemic, and potentially fill the current void of data on PD in patients with KC. Thereby, a potential influence of the COVID-19 pandemic on distress levels in urologic cancer patients will be delineated.

## Methods

### Study cohort & study design

All patients undergoing a 3-week IR between 01/2021 and 12/2021 in a specialized center (Center for Urological Rehabilitation, Kliniken Hartenstein, Bad Wildungen, Germany) after RP, RC, or (partial) nephrectomy were included in this study. In RC patients, the analysis focused on patients with the two most common types of urinary diversion, ileal conduit (IC) or ileal neobladder (INB). All patients gave their informed consent prior to data collection. The study was approved by the appropriate ethics board (2024–3675-evBO). Participants were routinely screened for PD by the validated Questionnaire on Stress in Cancer Patients – R23 (QSC–23) [20] at the beginning (T1) and end (T2) of IR. In the QSC–R23, a score ≥ 34 points signals high PD. Baseline clinical characteristics include age, sex, tumor stage, surgical approach, and for BC patients type of urinary diversion after RC.

### Inpatient rehabilitation

IR is firmly established in the German healthcare system and aims to support cancer patients to reach the important goal of reintegration into daily life after treatment. German social laws entitle cancer patients to an average of three weeks of IR, which has led to the establishment of dedicated centers for IR. Accordingly, German guidelines recommend IR after RP, RC, and (partial) nephrectomy, respectively, to minimize functional and psychological disorders and facilitate reintegration into working life for employed patients [21–23]. Calculations have shown that the cost of IR amortizes within 4 months due to saved (disability) pensions and collected taxes and contributions [24].

Psychosocial interventions for all patients are carried out by physicians, nurses, physiotherapists, psychologists, and social workers. The program includes information on cancer treatment and aftercare, individual, group, and couples psychotherapy, relaxation training, and psychoeducation. The multimodal therapy for patients after RP and RC includes, amongst other measures, osteopathic physiotherapy, external urethral sphincter exercises, and for patients with an INB or an IC after RC, educational training on INB management and stoma care, respectively. For patients after (partial) nephrectomy, IR consists of osteopathic physiotherapy, education on KC, chronic kidney disease, and sufficient fluid intake.

### Statistical analysis

Descriptive statistics for categorical variables included frequencies and proportions, while for continuous variables medians and interquartile ranges (IQR) were reported. Between-group comparisons were analyzed using the Mann–Whitney U test or Chi-square test (Pearson) as appropriate. The Wilcoxon test was used to compare changes in quantitative variables, while a Chi-squared test (McNemar) was used to compare changes in proportions. Multivariable logistic regression analyses were performed to identify predictors of high PD (QSC–R23 score ≥ 34). Significance was considered at *p* < 0.05. Data were analyzed by SPSS version 29.0 (IBM, Chicago, IL, USA).

## Results

### Patient characteristics

A total of 4,290 patients (3,413 after RP, 563 after RC, 314 after (partial) nephrectomy) were included in this study. Baseline characteristics of all patients categorized by malignancy can be found in Tables 1, 2, and 3.

**Table 1:** Baseline characteristics of 3,413 patients after radical prostatectomy

Variable	Total
Age, Median (IQR)	67 years (61–71)
PSA presurgical, Median (IQR)^a^	7.6 ng/ml (5.5–11.1)
Robot-assisted approach	72.0%
Tumor stage ≥ pT3^b^	38.9%
Lymph node positive^c^	10.8%
Positive surgical margin (R1)^d^	18.6%
GS ≥ 8^e^	17.4%

Abbreviations: *IQR* interquartile range, *PSA* prostate specific antigen, *GS* Gleason score

^a^data available for *n* = 3234

^b^data available for *n* = 3371

^c^data available for *n* = 3025

^d^data available for *n* = 3322

^e^data available for *n* = 3344

**Table 2:** Baseline characteristics of 563 patients after radical cystectomy

Variable	Total	IC (*n* = 328)	INB (*n* = 235)	*p*
Age, Median (IQR)	68 years (61–74)	71 years (65–77)	63 years (57–68.5)	** < 0.001**
Male sex	79.8%	52.6%	47.4%	** < 0.001**
Female sex	20.2%	80.7%	19.3%	** < 0.001**
Tumor stage ≥ pT3^a^	32.4%	41.4%	20.3%	** < 0.001**
Lymph node positive^b^	18.2%	21.8%	13.3%	**0.011**

Abbreviations: *IC* ileal conduit, *INB* ileal neobladder, *IQR* interquartile range

^a^data available for *n* = 552

^b^data available for *n* = 533

Bold font indicates significant results

**Table 3:** Baseline characteristics of 314 patients after (partial) nephrectomy

Variable	Total
Age, Median (IQR)	66 years (58–74)
Male sex	69.7%
Open surgery	66.2%
Robot-assisted approach	25.5%
Laparoscopic surgery	8.3%
Partial nephrectomy	51.6%
Tumor stage^a^	
pT1	67.5%
pT2	9.6%
≥ pT3	22.9%

Abbreviations: *IQR* interquartile range

^a^data available for *n* = 292

IR in the RP cohort started a median of 19 days (interquartile range (IQR) 15–22) after surgery. Median age in this cohort was 67 years (IQR 61–71). A tumor stage ≥ pT3 was found in 38.9%, positive surgical margins on final histopathology in 18.6%, and lymph node metastases in 10.8% of patients, respectively.

In the RC cohort (79.8% male, 20.2% female; 41.7% INB, 58.3% IC), IR started a median of 28 days (IQR 23–34) after surgery. Patients with an IC were significantly older (median age 71 years (IQR 65–77) vs. 63 years (IQR 57–68,5), *p* < 0.001) and suffered significantly more often from a locally advanced tumor stage (≥ pT3, 41.1% vs. 20.3%, *p* < 0.001) and lymph node metastases (21.8% vs. 13.3%, *p* < 0.011), respectively. Additionally, women suffered more often from tumor stage ≥ pT3 (43.9% vs. 29.5%, *p* = 0.003) and lymph node metastases (26.9% vs. 16.0%, *p* = 0.009) than their male counterparts.

For patients after (partial) nephrectomy (69.7% male), IR started a median of 20 days (IQR 17–23) after surgery. Tumor stage distribution was as follows: pT1: 67.5%, pT2: 9.6%, ≥ pT3: 22.9%. The majority of patients underwent open surgery (66.2%), while robot-assisted and laparoscopic surgery were performed in 25.5% and 8.3% of patients, respectively. Partial nephrectomy was performed in 51.6% of patients.

### Psychosocial distress according to type of malignancy (QSC–R23)

Data on the development of PD in general and high PD across the three disease entities can be found in Table 4.

**Table 4:** Development of psychosocial distress during inpatient rehabilitation

		Prostate cancer		Bladder cancer		Kidney cancer	
QSC-R23, Median (IQR)	T1	16 (9–30)	***p* < 0.001**	24 (13–39)	***p* < 0.001**	25 (13–39)	***p* < 0.001**
	T2	12 (6–24)		18 (7–32)		18 (9–35)	
High psychosocial distress (%)	T1	20.4%	***p* < 0.001**	32.9%	***p* < 0.001**	32.0%	*p* = 0.310
	T2	13.6%		22.4%		28.7%	

Abbreviations: *QSC-R23* questionnaire on stress in cancer patients, *IQR* interquartile range, *T1* start of inpatient rehabilitation, *T2* end of inpatient rehabilitation

Bold font indicates significant results

#### PD in patients after RP

The median QSC–R23 score in patients after RP decreased significantly from 16 points (IQR 9–30) at T1 to 12 points (IQR 6–24) at T2 (*p* < 0.001). High PD (QSC–R23 ≥ 34 points) was reported by 20.4% of patients at T1. The number of patients affected by high PD decreased significantly during IR to 13.6% at T2 (*p* < 0.001). Patient with tumor stage ≥ pT3 showed a significantly higher median QSC–R23 score (17 (IQR 9–31) vs. 16 (IQR 8–28), *p* = 0.007) and were significantly more often affected by high PD (22.2% vs. 19.0%, p = 0.032) than patients with tumor stage ≤ pT2. Furthermore, patients with positive surgical margins on final histopathology reported a higher median QSC–R23 score (19 (IQR 10–34) vs. 16 (IQR 8–28); *p* < 0.001) and were more often affected by high PD (25.3% vs. 19.1%, *p* < 0.001) than their counterparts without positive surgical margins. Moreover, patients with lymph node metastases reported a higher median QSC–R23 score (19 (IQR 11–33) vs. 16 (IQR 9–29), *p* = 0.002), were affected by high PD significantly more often (24.9% vs. 20.0%, *p* = 0.044), and significantly more often underwent individual psycho-oncologic counseling (39.0% vs. 31.5%, *p* = 0.006) than patients without lymph node metastases. A univariable logistic regression analysis identified younger age (odds ratio (OR) 0.944, 95% confidence interval (CI) 0.932 – 0.955, *p* < 0.001), tumor stage ≥ pT3 (OR 1.210; 95% CI 1.016–1.442, *p* = 0.032), positive surgical margins on final histopathology (OR 1.442, 95% CI 1.168–1.782, *p* < 0.001), and lymph node metastases (OR 1.328, 95% CI 1.007–1.752, *p* = 0.044) as predictors of high PD at T1. Meanwhile, multivariable logistic regression analysis identified younger age (OR 0.940, 95% CI 0.928–0.953, *p* < 0.001) and positive surgical margins on final histopathology (OR 1.362, 95% CI 1.060–1.750, *p* = 0.016) as independent predictors of high PD at this point. Tumor stage ≥ pT3, lymph node metastases, and presurgical PSA levels did not predict high PD (Table 5). Interestingly, the demand for individual psycho-oncologic counseling in patients after RP was significantly elevated compared to data obtained in our institution in 2019 (2019: 25.9% vs. 2021: 32.3%, *p* = 0.03).

**Table 5:** Uni- and multivariable logistic regression analysis to identify predictors of high psychosocial distress at the beginning of inpatient rehabilitation after radical prostatectomy for prostate cancer

(a)	Univariable		Multivariable	
	OR (95% CI)	*p*	OR (95% CI)	*p*
Age (continuous)	0.944 (0.932–0.955)	** < 0.001**	0.940 (0.928–0.953)	** < 0.001**
Tumor stage ≥ pT3	1.210 (1.016–1.442)	**0.032**	1.146 (0.922–1.424)	0.220
Positive surgical margins	1.442 (1.168–1.782)	** < 0.001**	1.362 (1.060–1.750)	**0.016**
Lymph node metastases	1.328 (1.007–1.752)	**0.044**	1.160 (0.842–1.600)	0.364
Baseline PSA (continuous)	1.002 (1.000–1.005)	0.088	1.002 (0.999–1.005)	0.168

Abbreviations: *OR* odds ratio, *CI* confidence interval, *PSA* prostate specific antigen

Bold font indicates significant results

#### PD in patients after RC

In patients after RC, a significant decrease in PD was observed between T1 and T2 (median QSC–R23 score 24 (IQR 13–39) vs. 18 points (IQR 7–32), *p* < 0.001). Furthermore, significantly fewer patients suffered from high PD at T2 (22.4%) than at T1 (32.9%, *p* < 0.001). No significant differences in PD levels were found when comparing patients with tumor stage ≥ pT3 to tumor stage ≤ pT2 and when comparing patients with to patients without lymph node metastases. Although the median QSC–R23 score did not differ significantly between patients with an IC and those with an INB (24 (IQR 13–40) vs. 23 (IQR 12.5–36), *p* = 0.223), patients with an IC were significantly more often impacted by high PD (37.0% vs. 27.4%, *p* = 0.024). Furthermore, female patients were significantly more likely to undergo individual psycho-oncological counseling than their male counterparts (48.2% vs. 32.5%, *p* = 0.002). Younger age (OR 0.969, 95% CI 0.950–0.989, *p* < 0.002) and IC (OR 1.554, 95% CI 1.060–2.280, *p* = 0.024) were identified as predictors of high PD at T1. Both findings were confirmed by multivariable linear regression analysis, which identified younger age (OR 0.948, 95% CI 0.925–0.970, *p* < 0.001) and IC (OR 2.346, 95% CI 1.469–3.745, *p* < 0.001) as independent predictors of high PD. Meanwhile, tumor stage ≥ pT3, female sex, and lymph node metastases did not predict high PD at this point (Table 6). In patients after RC, no increase in demand for individual psycho-oncologic counseling could be detected when compared to data from 2019 (37.1% vs. 37.3%, *p* = 0.92).

**Table 6:** Uni- and multivariable logistic regression analysis to identify predictors of high psychosocial distress at the beginning of inpatient rehabilitation after radical cystectomy for bladder cancer

(b)	Univariable		Multivariable	
	OR (95% CI)	*p*	OR (95% CI)	*p*
Age (continuous)	0.969 (0.950–0.989)	**0.002**	0.948 (0.925–0.970)	** < 0.001**
Tumor stage ≥ pT3	1.369 (0.922–2.033)	0.119	1.300 (0.830–2.037)	0.252
Female sex	1.042 (0.659–1.645)	0.861	0.892 (0.543–1.467)	0.653
Lymph node metastases	1.221 (0.757–1.968)	0.413	1.088 (0.642–1.844)	0.755
Ileal conduit	1.554 (1.060–2.280)	**0.024**	2.346 (1.469–3.745)	** < 0.001**

Abbreviations: *OR* odds ratio, *CI* confidence interval

Bold font indicates significant results

#### PD in patients after (partial) nephrectomy

Analog to patients after RP or RC, median PD in patients with KC decreased significantly during IR (median QSC–R23 score 25 (IQR 13–39) at T1 vs. 18 (IQR 9–35) at T2; *p* < 0.001). However, no significant decrease in patients suffering from high PD could be observed (32.0% at T1 vs. 28.7% at T2, *p* = 0.310). Patients after nephrectomy were more often affected by high PD (38.3% vs. 26.5%, *p* = 0.037), while patients after partial nephrectomy underwent individual psycho-oncologic counseling significantly more often (46.3% vs. 34.2%, *p* = 0.029). No difference in PD levels, in the percentage of patients suffering from high PD, and the demand for individual psycho-oncologic counseling were found between the sexes. When stratifying by tumor stage (pT1 vs. ≥ pT2), no significant differences in median QSC–R23 scores (26 (IQR 13–42) vs. 23.5 (IQR 13–35); *p* = 0.330) and patients suffering from high PD (33.1% vs. 29.3%, *p* = 0.535) were detected. Nonetheless, patients with tumor stage pT1 significantly more often underwent individual psycho-oncologic counseling than patients with tumor stage ≥ pT2 (49.2% vs. 27.4%, *p* < 0.001). Univariable logistic regression analysis identified younger age (OR 0.965, 95% CI 0.942–0.990, *p* < 0.005) and nephrectomy (OR 1.718, 95% CI 1.030–2.863, *p* = 0.038) as predictors of PD. These findings were confirmed by multivariable logistic regression analysis, which identified younger age (OR 0.958, 95% CI 0.933–0.984, *p* = 0.002) and nephrectomy (OR 1.973, 95% CI 1.058–3.677, *p* = 0.032) as independent predictors of high PD at T1. Tumor stage ≥ pT3, female sex, and open surgery were not found to significantly contribute to PD after (partial) nephrectomy (Table 7). Similar to observations made in patients after RP, the demand for individual psycho-oncologic counseling in patients with KC increased significantly from 26.9% in 2019 to 40.4% in 2021 (*p* = 0.001).

**Table 7:** Uni- and multivariable logistic regression analysis to identify predictors of high psychosocial distress at the beginning of inpatient rehabilitation after (partial) nephrectomy for kidney cancer

(c)	Univariable		Multivariable	
	OR (95% CI)	*p*	OR (95% CI)	*p*
Age (continuous)	0.965 (0.942–0.990)	**0.005**	0.958 (0.933–0.984)	**0.002**
Tumor stage ≥ pT3	0.883 (0.465–1.674)	0.702	0.592 (0.278–1.258)	0.173
Female sex	0.828 (0.468–1.463)	0.515	0.888 (0.481–1.642)	0.706
Nephrectomy	1.718 (1.030–2.863)	**0.038**	1.973 (1.058–3.677)	**0.032**
Open surgery	1.440 (0.830–2.498)	0.194	1.636 (0.887–3.016)	0.115

Abbreviations: *OR* odds ratio, *CI* confidence interval

Bold font indicates significant results

## Discussion

Our study shows a high prevalence of PD in patients after RP, RC, or (partial) nephrectomy. The key finding of our study is that younger age, independent of the investigated malignancy, predicts high PD in the early period after surgery. Furthermore, disease-specific independent predictors (positive surgical margins on final histopathology in PC patients, IC in RC patients, nephrectomy in KC patients) are identified. Although PD decreased significantly during IR, about 14% of PC patients, 22% of BC patients, and 29% of KC patients reported high PD at the end of IR respectively. For PC, these findings are in line with previous retrospective data by our group [9]. Complications after RP such lymphoceles may contribute to PD and could potentially be reduced by more individually tailored approaches such as PSMA radio-guided surgery [25] or unilateral lymph node dissection [26]. However, both approaches are not established yet.

The proportion of patients with high PD decreases significantly in PC and RC patients during IR, but not so in KC patients. In patients after RC, this study corroborates previous findings of patients with an IC suffering from locally advanced disease and lymph node metastases more often than patients with an INB and decreasing PD during IR after RC independent of the type of urinary diversion [10]. Tackling PD as early as possible is underscored by an increase and long-term persistence of high PD in a considerable number of patients after RC [27, 28]. Complications in the early period after RC are common [29] and impact quality of life [30]. However, their influence on PD has not been investigated yet. Studies investigating this topic are therefore needed.

The aforementioned studies by Draeger et al. and Bergerot et al. on PD in KC patients identified anxiety, pain, nervousness, sadness, and sleep difficulties as contributors to PD in general and female sex, younger age, non-clear cell histology, and recurrent disease as predictors for high PD, respectively [13, 14]. However, both cohorts included patients with localized and metastatic KC and used the National Comprehensive Cancer Network Distress Thermometer and Hornheider Screening Instrument, as opposed to a cohort with unexceptionally surgical treatment and screening by the QSC–R23 in our study. Pastore et al. performed a cross-sectional study in 207 patients with BC (*n* = 125), PC (*n* = 50), or KC (*n* = 15) and identified significantly higher PD in patients with KC scheduled for nephrectomy [15]. However, the small number of patients with KC included in this study is a strong limitation and all patients underwent nephrectomy. Furthermore, patients undergoing transurethral resection of a bladder tumor made up the majority of the cohort (52.7%), as opposed to our BC cohort exclusively made up of patients after RC. Therefore, a comparison of the results to those of our study is of limited value. Moreover, all three studies investigating PD in KC patients only evaluated PD at a single time point (e.g., before surgery or start of medical treatment) and not longitudinally. Consequently, the influence of confounders on the results cannot be ruled out. Interestingly, at the single point evaluation, neither study reported higher PD in patients with metastatic disease, although it has to be mentioned that only 3 patients undergoing medical treatment were included in the study by Draeger et al. In contrast to our results, Ajaj et al. identified elevated distress levels in female patients and lower distress levels in younger patients in the early period after surgery, respectively [31]. However, the Edmonton Symptom Assessment System – revised (ESAS-r) questionnaire used in their study was originally developed to assess symptoms in patients undergoing palliative care and only includes two item assessing psychological wellbeing. Meanwhile, the QSC–R23 used in our study was specifically developed to only screen PD in cancer patients and therefore provides a more comprehensive assessment.

Alarmingly, the suicide rate in patients with PC, BC, or KC is significantly higher than in the general population [32, 33], Increasing age and metastasized disease are associated with a higher probability of suicide, highlighting the need for intensified screening and treatment of PD in cancer patients.

Limitations of our study include the lack of a control group for each disease entity outside of IR and, due to the study design, missing baseline levels of PD. Furthermore, surgical complications and the prevention of these by the routine implementation of a protocol for enhanced recovery after surgery (ERAS) in the primary hospitals could not be assessed. Of note, the data discussed in this paper were obtained during the COVID-19 pandemic, so the influence of potentially elevated “baseline distress” levels on our results due to the pandemic cannot be ruled out completely. This assumption is further supported by a significantly increased demand for individual psycho–oncologic counseling in patients with PC or KC when compared to pre-pandemic data from our institution, potentially mirroring increasing distress levels and mental health issues during the COVID-19 pandemic in both the general population and cancer patients [19]. As of now, the long-term impact of the COVID-19 pandemic on the mental health of cancer survivors remains to be determined.

To our knowledge, this is the first study to investigate PD in a large and multi-institutional German cohort exclusively consisting of patients after surgical treatment of the three most common urological malignancies (PC, BC, KC). Our study provides comprehensive analyses of PD in the three main genitourinary malignancies in a large patient cohort recruited in a short and recent period. IR poses a unique opportunity to analyze patients from all over Germany and all levels of care, providing real-world data and thereby facilitating the development of evidence-based decision pathways.

## Conclusion

PD is prevalent among all three urological cancers investigated in this study, again highlighting the need for intensified psycho-oncological counseling in the aftercare of patients after RP, RC, and (partial) nephrectomy. Disease-specific predictors for high PD may help clinicians identify patients at risk and may guide timely referrals to psycho-oncologic counseling in the early period after RP, RC, or (partial) nephrectomy.

## Data Availability

No datasets were generated or analysed during the current study.
